# Loss of Tdp-43 disrupts the axonal transcriptome of motoneurons accompanied by impaired axonal translation and mitochondria function

**DOI:** 10.1186/s40478-020-00987-6

**Published:** 2020-07-24

**Authors:** Michael Briese, Lena Saal-Bauernschubert, Patrick Lüningschrör, Mehri Moradi, Benjamin Dombert, Verena Surrey, Silke Appenzeller, Chunchu Deng, Sibylle Jablonka, Michael Sendtner

**Affiliations:** 1 Institute of Clinical Neurobiology, University of Wuerzburg, 97078 Wuerzburg, Germany; 2 Comprehensive Cancer Center Mainfranken, University of Wuerzburg, 97080 Wuerzburg, Germany

**Keywords:** Amyotrophic lateral sclerosis, Tdp-43, Axonal transcriptome, Nicotinamide

## Abstract

Protein inclusions containing the RNA-binding protein TDP-43 are a pathological hallmark of amyotrophic lateral sclerosis and other neurodegenerative disorders. The loss of TDP-43 function that is associated with these inclusions affects post-transcriptional processing of RNAs in multiple ways including pre-mRNA splicing, nucleocytoplasmic transport, modulation of mRNA stability and translation. In contrast, less is known about the role of TDP-43 in axonal RNA metabolism in motoneurons. Here we show that depletion of Tdp-43 in primary motoneurons affects axon growth. This defect is accompanied by subcellular transcriptome alterations in the axonal and somatodendritic compartment. The axonal localization of transcripts encoding components of the cytoskeleton, the translational machinery and transcripts involved in mitochondrial energy metabolism were particularly affected by loss of Tdp-43. Accordingly, we observed reduced protein synthesis and disturbed mitochondrial functions in axons of Tdp-43-depleted motoneurons. Treatment with nicotinamide rescued the axon growth defect associated with loss of Tdp-43. These results show that Tdp-43 depletion in motoneurons affects several pathways integral to axon health indicating that loss of TDP-43 function could thus make a major contribution to axonal pathomechanisms in ALS.

## Introduction

In neurons, mechanisms for subcellular mRNA transport and translation in dendrites and axons allow the regulation of localized responses to cellular stress conditions. Alterations in these RNA processing mechanisms can lead to neural dysfunction and neurodegeneration [1–3]. Likewise, disturbed subcellular RNA distribution has been found to be a pathophysiological hallmark in amyotrophic lateral sclerosis (ALS) [4–6] and spinal muscular atrophy (SMA) [7].

Subcellular mRNA transport is mediated by messenger ribonucleoprotein particles (mRNPs) which contain RNA-binding proteins as essential components. One of these RNA-binding proteins is the transactive response DNA-binding protein (TARDBP or TDP-43) [4]. Initially, TDP-43 was identified as a strong transcriptional repressor of human immunodeficiency virus gene expression by binding to the TAR DNA in transfected cells [8]. Since then, multiple roles in RNA processing have been identified for TDP-43 such as regulation of pre-mRNA splicing, translation, miRNA processing, mRNA stability and subcellular transport [9–11]. Under physiological conditions, TDP-43 is predominantly found in the nucleus although the protein is able to shuttle between the nucleus and the cytoplasm [12], suggestive of a role in mRNA stabilization and mRNP trafficking from the nucleus to the cytoplasm, in addition to its nuclear function in transcriptional regulation.

ALS pathology is generally characterized by degeneration of upper and lower motoneurons, resulting in a dramatic loss of motor function [13]. Disruption of axonal RNA transport might contribute to the specific pathophysiology in motoneurons [14]. For example, the motility of axonal TDP-43 transport granules is impaired when mutant TDP-43 harboring ALS-associated mutations is expressed in neurons [4]. In line with this finding, the anterograde axonal transport of TDP-43-associated mRNA granules is affected in induced pluripotent stem (iPS) cell-derived motoneurons from ALS patients [4]. Axonal transport defects have also been reported in Drosophila models of ALS [15], suggesting a conserved cellular function of TDP-43 in subcellular localization of RNA. RNA immunoprecipitation sequencing (RIP-seq) and crosslinking and immunoprecipitation (CLIP) studies revealed specific binding sites of TDP-43 along single transcripts and identified transcript level changes of several mRNAs after TDP-43 depletion [16–18]. Preferential binding of TDP-43 to the 3´UTRs of specific mRNAs was found for cytoplasmically localized TDP-43, which might be reflective of its role in subcellular mRNA localization [18].

In order to investigate how TDP-43 is involved in modulation of axonal functions, we determined the consequences of Tdp-43 depletion in primary mouse motoneurons both on the morphological level and by analysis of alterations in the somatodendritic and axonal transcriptomes. Tdp-43 knockdown significantly impaired axon elongation. RNA-seq analysis revealed that many transcripts relevant for axonal cytoskeletal integrity, mitochondrial function and translation were dysregulated in the axonal compartment upon Tdp-43 depletion. In agreement with this finding, protein translation and mitochondrial functionality were specifically impaired in axons of Tdp-43-deficient motoneurons. Finally, we found that treatment with nicotinamide, a precursor for the cofactor NAD+ that is required for oxidative phosphorylation, restored axon growth in Tdp-43 knockdown motoneurons to normal levels. Our results therefore suggest that reduction of Tdp-43 function dysregulates RNA transport processes that are vital for axon growth, local protein synthesis and energy production.

## Materials and methods

### Animals

CD-1 mice were housed in the animal facility of the Institute of Clinical Neurobiology at the University Hospital of Wuerzburg. Mice were maintained at a 12 h/12 h day/night cycle under controlled conditions in 55–65% humidity. Food and water were available in abundant supply. Each experiment was conducted following the regulations on animal protection of the German federal law and of the Association for Assessment and Accreditation of Laboratory Animal Care, in agreement with and under control of the local veterinary authority.

### Isolation and culture of primary embryonic mouse motoneurons

Spinal cords were dissected from E12.5 CD-1 mouse embryos as described previously [19]. Dorsal root ganglia and meninges were removed and tissue was digested by 0.1% trypsin (Worthington). Motoneurons were enriched by incubation with anti-p75 antibody-coated (MLR2, Abcam) cell culture dishes. For axon length analysis, 2000 cells were plated per glass coverslip and cultured in the presence of BDNF (5 ng/ml) in neurobasal medium (Invitrogen) containing 500 μM GlutaMAX (Invitrogen), 2% horse serum (Gibco) as well as 2% B27 supplement (Invitrogen) for 7 d at 37 °C with 5% CO_2_. 50% of the culture medium was replaced at 1 d in vitro (DIV1) and then every second day. For nicotinamide treatment, medium was supplemented with nicotinamide (Applichem) at the indicated concentrations. Motoneuron cultures in compartmentalized chambers were performed as previously described [7]. CNTF was used at a concentration of 5 ng/ml on the somatodendritic and axonal side and BDNF at a concentration of 20 ng/ml on the axonal side.

### Knockdown of Tdp-43 via lentiviral shRNA in embryonic mouse motoneurons

Four different siRNA sequences targeting *Tdp-43* were designed using the siRNA Wizard (Invivogen). The oligonucleotides for cloning the corresponding shRNAs into the pSIH-H1 shRNA vector (System Bioscience) are listed in Supplementary Table 1. All vectors generated in this study were sequenced in order to verify their integrity. HEK293T cells were used for virus production as described previously [20]. For lentiviral transduction, motoneurons were incubated for 10 min at room temperature (RT) with knockdown or control virus prior to plating. *Tdp-43* transcript levels were measured by qPCR using *Gapdh* as housekeeping transcript.

### Immunofluorescence staining of free-floating spinal cord sections

Free-floating sections were essentially prepared as described before [21]. Mice were transcardially perfused with 4% paraformaldehyde (PFA). Subsequently, the spinal cord was removed and post-fixed in 4% PFA for 2 h at RT. After embedding in 5% agarose, 40 μm thick, free-floating sections were cut on a Leica VT1000 S microtome and collected in 0.1 M phosphate buffer pH 7.4. After incubation for 2 h at RT with blocking solution [10% donkey serum, 0.3% Triton X-100 and 0.1% Tween-20 in Tris-buffered saline (TBS)], the sections were incubated for 72 h at 4 °C with primary antibodies in 1:10 diluted blocking solution (1% donkey serum, 0.03% Triton X-100 and 0.01% Tween-20 in TBS). After washing three times for 10 min in TBS-Tween (TBS-T) at RT, sections were incubated for 2 h at RT with the appropriately fluorophore-conjugated secondary antibodies, washed again in TBS-T and finally mounted with Fluor Save (Merck-Millipore). The following primary antibodies were used: polyclonal goat anti-ChAT (MAB144P, Millipore; 1:1000) and polyclonal rabbit anti-TDP43 (10782–2-AP, Proteintech; 1:1000).

### Immunofluorescence staining of cultured motoneurons

Motoneurons were washed with phosphate-buffered saline (PBS) and fixed with 4% PFA for 15 min at RT. For permeabilization, 0.3% Triton X-100 was applied for 20 min at RT. Motoneurons were treated with 2% donkey serum, 2% BSA, 2% saponin and 5% sucrose in PBS for at least 1 h to reduce unspecific binding followed by primary antibody incubation overnight at 4 °C. Motoneurons were washed thrice and incubated with secondary antibodies for 1 h at RT. Nuclei were visualized by DAPI and motoneurons were embedded with Aqua Poly/Mount (18606, Polysciences). The following primary and secondary antibodies were used for immunostaining: polyclonal rabbit anti-TDP-43 (10782–2-AP, Proteintech; 1:300), monoclonal mouse anti-α-Tubulin (T5168, Sigma-Aldrich; 1:1000), donkey anti-rabbit (H + L) IgG (Cy3; 711–165-152, Jackson Immunoresearch; 1:500) and goat anti-mouse (H + L) IgG (Cy5; 115–175-146, Jackson Immunoresearch; 1:500).

### Axon length measurements

Motoneurons transduced with lentiviruses were immunostained at DIV7 with rabbit polyclonal anti-Tau (T6402, Sigma-Aldrich; 1:800) and chicken polyclonal anti-GFP (ab13970, Abcam; 1:2000) antibodies. The length of the longest axon branch was measured using ImageJ software; axon collaterals were not considered for the analysis. Motoneurons were only scored when designated axons were at least three times longer than the corresponding dendrites ensuring an unambiguous distinction between axons and dendrites. Statistical analysis was performed using GraphPad Prism software (GraphPad Software).

### RNA-seq analysis

Whole transcriptome amplification and high-throughput sequencing of RNA from compartmentalized motoneurons were performed as previously described [22]. Briefly, total RNA was extracted from the somatodendritic and the axonal compartment of compartmentalized motoneurons and reverse-transcribed. Following second strand synthesis, fragments were PCR amplified and converted into high-throughput sequencing libraries. Sequencing, read mapping and data analysis were performed as described before [22]. Control datasets from motoneurons transduced with empty lentiviral expression vector were described before [1]. Control and Tdp-43 knockdown motoneurons were processed in parallel for RNA-seq. The sequencing data described in this publication are accessible in NCBI’s Gene Expression Omnibus [23] through GEO Series accession number GSE147607. For comparison with TDP-43 iCLIP, we used iCLIP data from control subjects from Tollervey et al. [18]. For GO term analysis we used the Database for Annotation, Visualization and Integrated Discovery (DAVID) [24]. As background datasets, we used the 10,433 and 11,127 transcripts detected in the somatodendritic and axonal compartment, respectively, of wildtype motoneurons [22].

### Puromycin labeling

After 6 DIV, primary motoneurons were labeled with puromycin (10 μg/ml) for 15 min. After 15 min, the cells were washed once with Hanks’ Balanced Salt Solution (HBSS) (Gibco), and fresh medium was added. The puromycin incorporation was chased for 45 min. For depolymerization of microtubules, cells were treated with 10 μM nocodazole for 2 h prior to puromycin labeling. For inhibition of protein synthesis, cultured motoneurons were treated with cycloheximide (10 μg/ml) or anisomycin (100 ng/ml) for 1 h prior to puromycin labeling. Subsequently, the cells were fixed with 2% PFA for 15 min at RT. Puromycin incorporation was visualized with an anti-puromycin antibody (clone 12D10, MABE343, Merck Millipore; 1:1000). Motoneurons were counterstained with an anti-Tau antibody (T6402, Sigma-Aldrich; 1:500).

### Measuring mitochondrial membrane potential in live mouse motoneurons

Mitochondria were labeled with MitoTracker™ Orange CM-H2TMRos (M7511, Thermo Fisher Scientific) and TMRM (Tetramethylrhodamine, Methyl Ester, Perchlorate, T668, Thermo Fisher Scientific) was used to determine the mitochondrial membrane potential (Δψm). Motoneurons were cultured on laminin-111-coated (23017–015, Invitrogen) 35 mm Ibidi μ-dishes (81,156, Ibidi) for 6 d. Cells were washed twice with pre-warmed Tyrode’s buffer [125 mM NaCl, 2 mM KCl, 30 mM glucose, 2 mM CaCl_2_, 2 mM MgCl_2_, and 25 mM HEPES (pH 7.4)] and 20 nM TMRM or MitoTracker™ Orange were loaded into cells by incubation for 15 min at 37 °C and 5% CO_2_. Then, motoneurons were washed twice with pre-warmed Tyrode’s buffer and imaged in 2 ml Tyrode’s buffer supplemented with 5 ng/ml BDNF. Live-cell imaging was conducted on an inverted epifluorescence microscope (TE2000, Nikon) equipped with 60× 1.4-NA objective, Perfect Focus System (Nikon), a Cool LED pE-4000 light source and an Orca Flash 4.0 V2 camera (Hamamatsu Photonics). During live-cell imaging, cells were maintained at 37 °C and 5% CO_2_ on a heated stage chamber (TOKAI HIT CO., LTD). TMRM and MitoTracker™ Orange CM-H2TMRos were excited at 560 nm illumination using 2% light intensity and 12-bit images of 1024 × 1024 pixels were acquired for 5 min at 30 s intervals. Mitochondria labeled with MitoTracker™ Orange were counted in axons of control and Tdp-43 knockdown motoneurons and normalized to axon length. ImageJ was used to measure the mean grey value of TMRM fluorescence intensity per mitochondrion and average background intensity was subtracted. Median intensity values of mitochondria per axon were normalized to the average intensity of the corresponding control group. In control experiments with FCCP, mitochondria labeled with TMRM were imaged first for 1 min with 30 s intervals. Next, 10 μM FCCP (C2920, Merck) was added and axons were imaged for another 8 min. FCCP depolarizes the mitochondrial membrane potential, resulting in a rapid and significant decrease in the fluorescent intensity of TMRM upon 1 min addition.

### qPCR

RNA was reverse-transcribed with random hexamers using the First Strand cDNA Synthesis Kit (Thermo Fisher Scientific). Reverse transcription reactions were diluted 1:5 in water. Reactions were set up with Luminaris HiGreen qPCR Master Mix (Thermo Fisher Scientific) on a LightCycler® 96 thermal cycler (Roche). Primers are listed in Supplementary Table 2.

## Results

### Tdp-43 protein localizes to axons and growth cones of primary mouse motoneurons and regulates axon growth

**Fig. 1:**
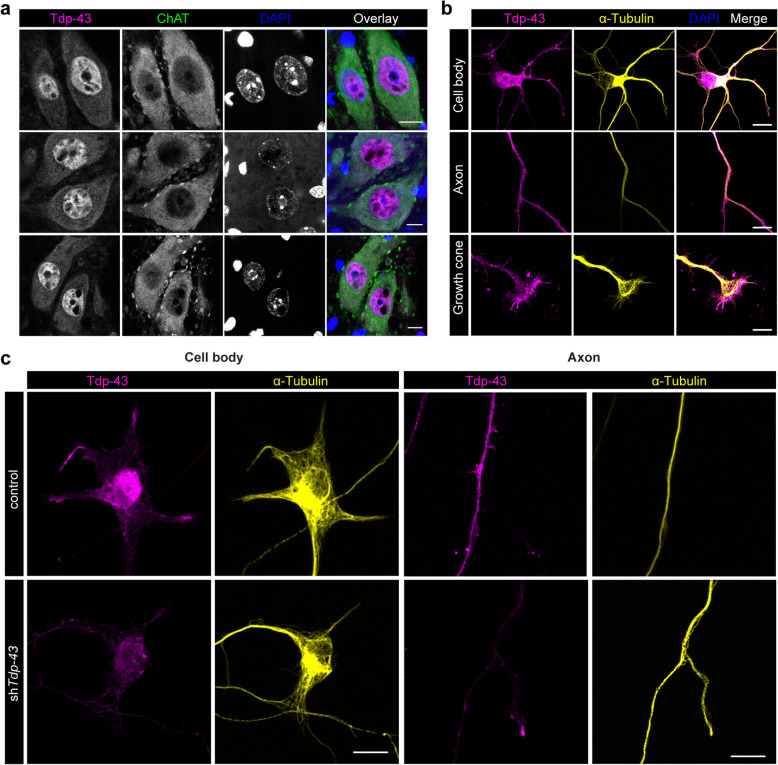
Tdp-43 localizes in the nucleus and cytosol of motoneurons. **a** Spinal cord cross sections of adult CD-1 mice immunostained with an antibody against Tdp-43 and an antibody against choline acetyltransferase (ChAT) as a motoneuron marker. DAPI was used for visualization of nuclei. Scale bar: 10 μm. **b** Primary mouse motoneurons (DIV6) were immunostained with an antibody against Tdp-43 and with an antibody against α-Tubulin. DAPI was used for visualization of nuclei. Scale bar: 10 μm. **c** Immunostaining of motoneurons transduced with empty lentiviral expression vector (control) or vector expressing a shRNA targeting *Tdp-43* (sh*Tdp-43*) with antibodies against Tdp-43 and Tubulin. Scale bar: 10 μm

In order to characterize the subcellular distribution and in particular the location and function of Tdp-43 in axons we first investigated the Tdp-43 distribution in spinal cord of adult mice by immunostaining (Fig. 1a). An antibody against acetylcholine transferase (ChAT) was used to identify motoneurons. In all ChAT-positive cells, Tdp-43 was prominently detectable both in the nucleus and the cytoplasm. To confirm this observation, we investigated the distribution of Tdp-43 in primary mouse motoneurons cultured for 6 d (Fig. 1b). An antibody against α-Tubulin was used for staining of axons. Tdp-43 immunoreactivity was detectable in the nucleus and in the cytoplasm, including axons, of motoneurons. Interestingly, Tdp-43 protein was not distributed in a diffuse manner but localized to distinct axonal structures. As a specificity control, these structures remained unlabelled when Tdp-43 expression was suppressed by shRNA expression (Fig. 1c, see below for details). This staining pattern resembled the axonal Tdp-43 distribution reported previously [25]. Positive structures in the axon probably include mitochondria that have been identified to bind TDP-43 [26, 27] but possibly also particles for axonal RNA transport and processing [4, 25]. Interestingly, such Tdp-43-positive spot-like structures appeared highly enriched in axonal growth cones of primary motoneurons (Fig. 1b).

**Fig. 2:**
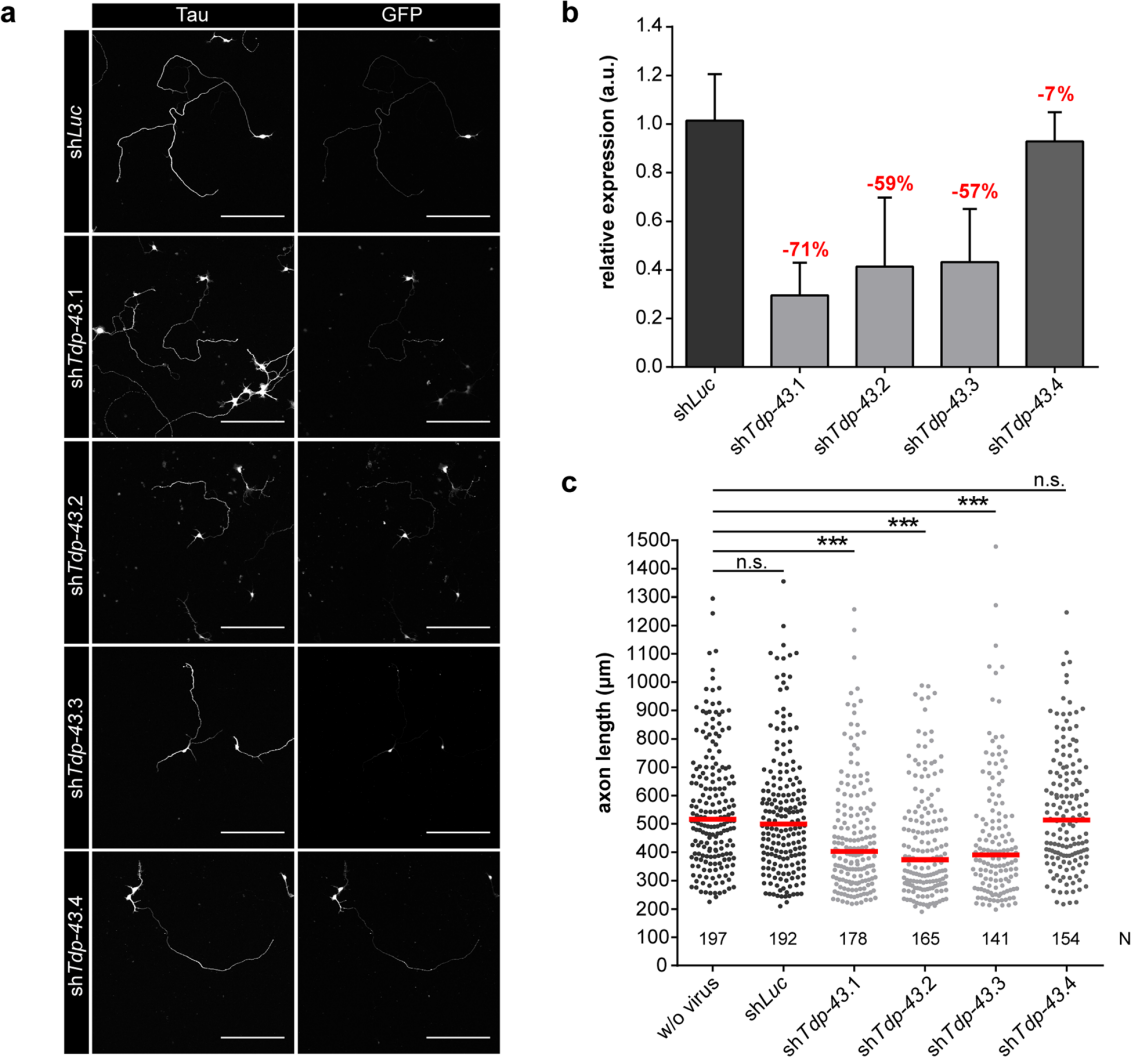
Tdp-43 regulates axon growth of motoneurons. **a** Immunostaining of motoneurons transduced with a control (sh*Luc*) or different Tdp-43 knockdown (sh*Tdp-43*) lentiviruses at DIV7 with antibodies against Tau and GFP. Scale bar: 200 μm. **b** Quantification of *Tdp-43* transcript levels in motoneurons transduced with shRNAs targeting *Tdp-43*. Bar graphs show *Tdp-43* transcript levels relative to control motoneurons transduced with sh*Luc*. Data represent mean ± standard deviation (SD) (*n* = 4). **c** Scatter plot showing axon lengths of non-infected motoneurons (w/o virus), control motoneurons transduced with sh*Luc* and Tdp-43 knockdown motoneurons (sh*Tdp-43*). Horizontal lines represent median. ***p ≤ 0.001; Kruskal-Wallis-Test (N numbers of axons are shown in the graph, *n* = 3 independent experiments)

To elucidate the function of Tdp-43 in motoneurons, we measured axon growth of Tdp-43-depleted embryonic motoneurons that were cultured for 7 d. For this purpose, we designed four different shRNAs (sh*Tdp-43*.1-4) targeting the *Tdp-43* transcript and cloned them into a lentiviral expression vector. The lentiviral constructs also expressed GFP for visualization of virus infection. We transduced primary motoneurons with these different Tdp-43 knockdown viruses or a control virus expressing a *Luciferase* shRNA (sh*Luc*) and cultured them for 7 d (Fig. 2a). At this time point Tdp-43 knockdown lentiviruses expressing sh*Tdp-43*.1, 2 or 3 reduced *Tdp-43* transcript levels by 57–71% relative to controls (Fig. 2b). In contrast, Tdp-43 knockdown virus expressing sh*Tdp-43*.4 was not effective in reducing *Tdp-43* mRNA levels. We then investigated the effect of Tdp-43 knockdown on axon growth. Motoneurons were immunostained for the axonal marker protein Tau and axon length was measured at DIV7 (Fig. 2a, c). Axon lengths of motoneurons transduced with sh*Tdp-43*.1, 2 or 3 were significantly reduced relative to controls. In agreement with the qPCR results for measuring knockdown efficacy, transduction with sh*Tdp-43*.4 had no significant effect on axon outgrowth. As transduction with sh*Tdp-43*.2 showed the highest reduction in axon length, we proceeded with this shRNA (denoted ‘sh*Tdp-43*’ hereafter) for all the subsequent Tdp-43 knockdown experiments and used it also for confirmation of the specificity of the Tdp-43 immunolabeling procedure (Fig. 1c).

### Loss of Tdp-43 dysregulates the somatodendritic and axonal transcriptome

To find out whether the disturbed axonal elongation of Tdp-43-deficient motoneurons could be due to subcellular transcriptome alterations we performed RNA-seq on the somatodendritic and axonal compartment of Tdp-43 knockdown motoneurons grown in microfluidic chambers [7]. For this purpose, we cultured the motoneurons for 7 d in these specialized culture dishes and then separately isolated total RNA from the two compartments. Due to the low amount of RNA that can be extracted from the axonal compartment we used a previously established pre-amplification protocol for RNA-seq in order to obtain sufficient levels of cDNA from each compartment for high-throughput sequencing [22]. Furthermore, this protocol enabled us to analyze whole transcriptome changes without any selection for RNA subtypes, and therefore also included noncoding RNAs and ribosomal RNAs (rRNAs).

**Fig. 3:**
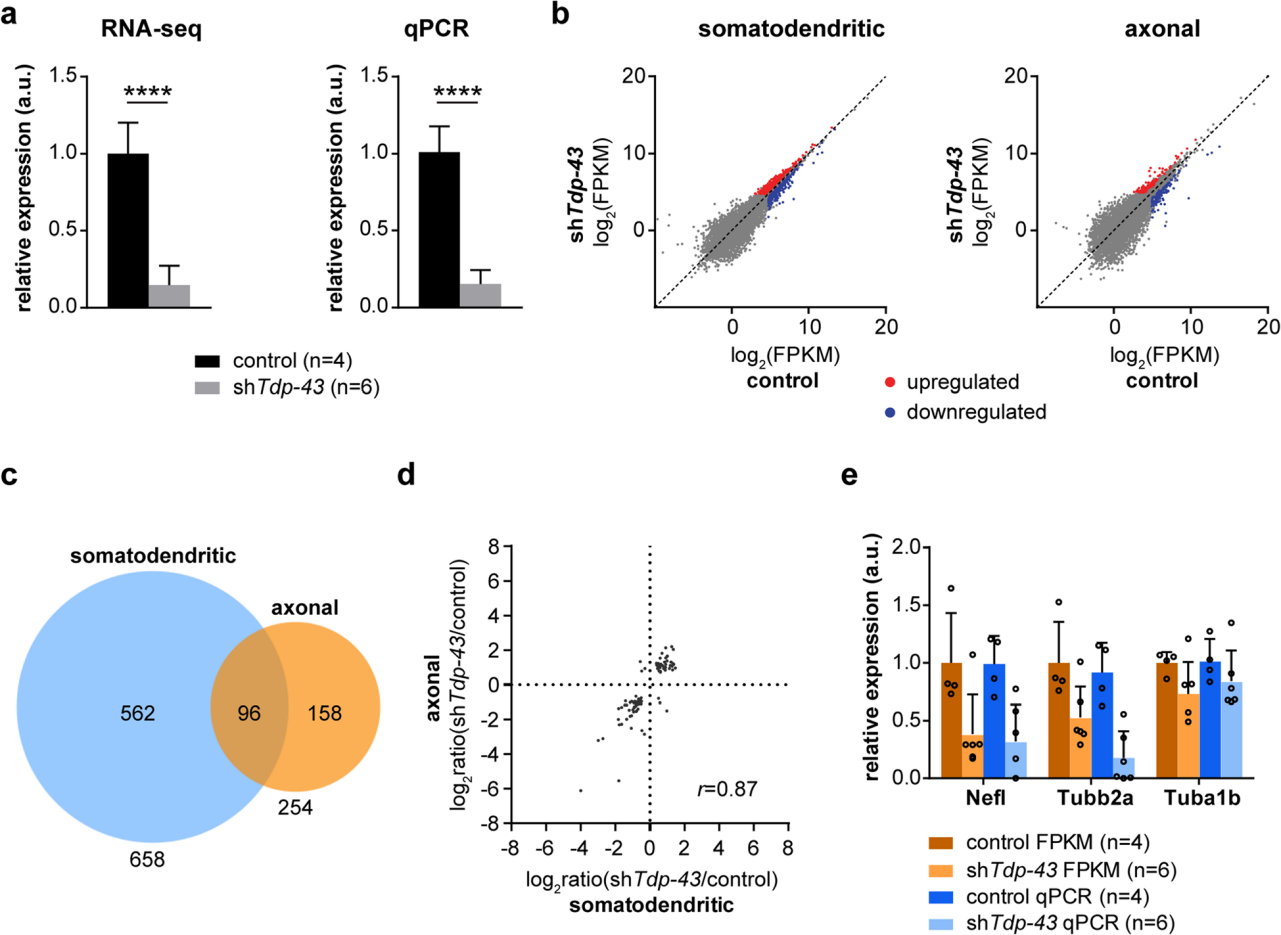
RNA-seq analysis of compartmentalized Tdp-43 knockdown motoneurons. **a** Bar graphs showing *Tdp-43* levels in Tdp-43 knockdown (sh*Tdp-43*) and control motoneurons transduced with empty lentiviral expression vector. Transcript levels were measured as FPKM values by RNA-seq analysis and by qPCR. Data represent mean ± SD. ****p ≤ 0.0001; unpaired t-test. **b** Scatter plots of somatodendritic and axonal transcript FPKM values following differential expression analysis of Tdp-43 knockdown motoneurons compared to controls. Transcripts significantly (*p* < 0.05) dysregulated in Tdp-43 knockdown motoneurons are colour-coded as red for upregulated and as blue for downregulated transcripts. **c** Venn diagram presenting the overlap of transcripts dysregulated in the somatodendritic and axonal compartment of Tdp-43 knockdown motoneurons. **d** Scatter plot depicting the logarithmized fold changes of the 96 transcripts altered in both the somatodendritic and axonal compartment of Tdp-43 knockdown motoneurons relative to controls. **e***Nefl*, *Tubb2a* and *Tuba1b* transcript levels in axons of Tdp-43 knockdown and control motoneurons measured by RNA-seq (as FPKM values) and qPCR. Data represent mean ± SD

We observed an 85% reduction of *Tdp-43* transcript levels by qPCR in Tdp-43 knockdown motoneurons transduced with sh*Tdp-43* relative to control motoneurons transduced with an empty lentiviral expression construct (Fig. 3a). Following RNA-seq a similar depletion of *Tdp-43* transcripts could be measured as FPKM (Fig. 3a). To identify transcripts dysregulated in the somatodendritic and axonal compartment of Tdp-43 knockdown motoneurons relative to control motoneurons we performed differential expression analysis (Fig. 3b). This revealed 658 transcripts that were significantly (*p* < 0.05) dysregulated in the somatodendritic compartment (Fig. 3c). Of these, 371 were upregulated (Supplementary Table 3) and 287 downregulated (Supplementary Table 4) upon Tdp-43 suppression. The *Tdp-43* transcript was the second most strongly decreased transcript which underscores the validity of our RNA-seq analysis. In the axonal compartment of Tdp-43 knockdown motoneurons, 254 transcripts were significantly dysregulated relative to controls (Fig. 3c). Of these, 118 transcripts were upregulated (Supplementary Table 5) and 136 downregulated (Supplementary Table 6). When we compared the dysregulated transcripts in the somatodendritic and axonal compartment, we identified a group of 96 transcripts that were significantly dysregulated in the axonal and somatodendritic compartments upon Tdp-43 loss (*p* < 0.05 in both axonal and somatodendritic compartment) (Supplementary Table 7) (Fig. 3c). Of these 96 transcripts, 47 transcripts were downregulated and 43 transcripts were upregulated in either compartment. The magnitude of change of these transcripts was highly correlated between the two compartments (Pearson’s *r* = 0.87) (Fig. 3d). These transcript alterations that are common to both compartments are likely due to global changes in transcription and/or stabilization, in line with functions of Tdp-43 in transcription and mRNA stability [9, 11].

We observed that several transcripts encoding for components of the cytoskeleton were downregulated. Among these were the transcripts encoding Neurofilament light chain polypeptide (Nefl), Tubulin beta-2A (Tubb2a) and Tubulin alpha-1B (Tuba1b). We validated the downregulation of these transcripts in axons of Tdp-43 knockdown motoneurons by qPCR (Fig. 3e). *Nefl* mRNA binds to TDP-43 and has been shown previously to be transported anterogradely as part of Tdp-43-containing granules in axons of mouse cortical neurons [4, 28]. Beyond that, *Nefl* mRNA levels are known to be reduced in degenerating motoneurons from ALS patients [29, 30]. Our result substantiates these findings by showing that loss of Tdp-43 in mouse motoneurons impairs axonal levels not only of *Nefl* but also of transcripts encoding other cytoskeletal components.

### Analysis of Tdp-43 binding on transcripts dysregulated by Tdp-43 deficiency

**Fig. 4:**
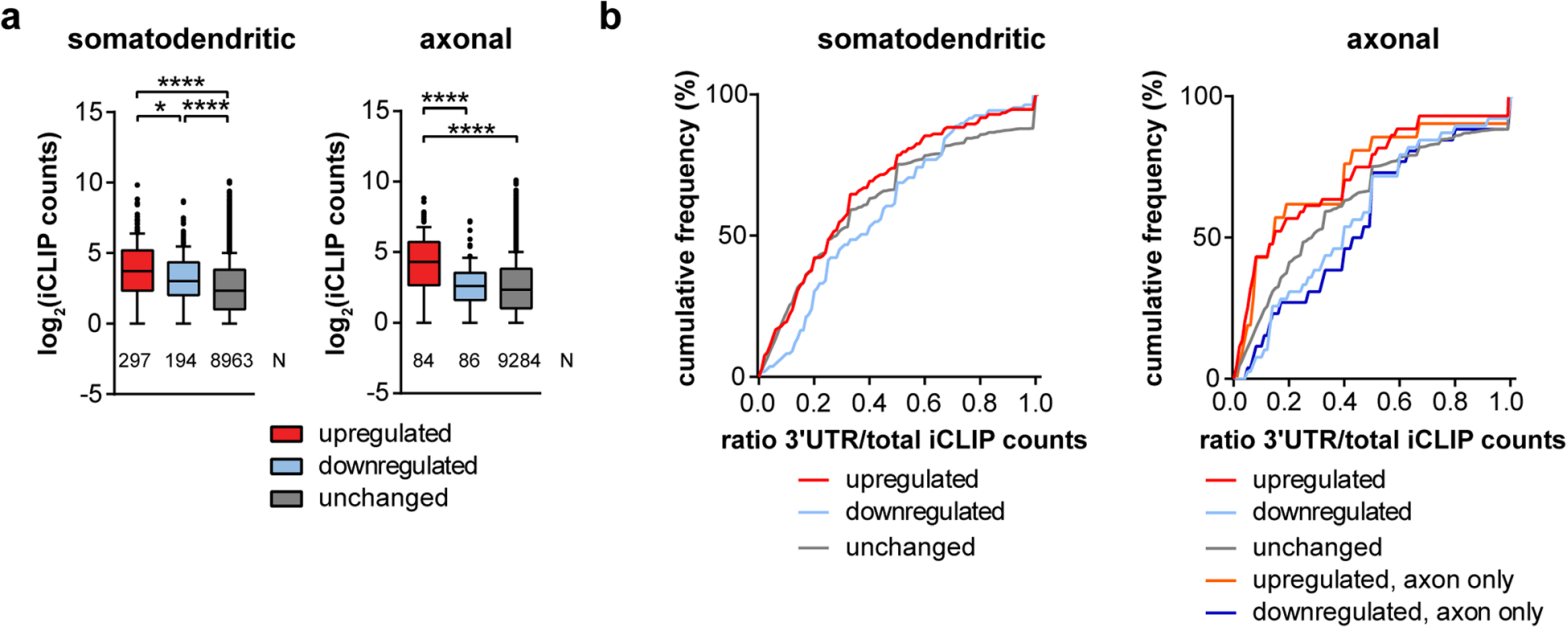
Analysis of TDP-43 binding to transcripts dysregulated in Tdp-43 knockdown motoneurons. **a** Boxplot showing the number of TDP-43 iCLIP counts [18] for upregulated, downregulated and unchanged transcripts in the somatodendritic and axonal compartment after Tdp-43 knockdown. Only transcripts with at least one iCLIP hit were included. *p ≤ 0.05, ****p ≤ 0.0001; Kruskal-Wallis test with Dunn’s multiple comparisons test (N numbers of transcripts are shown in the graph). **b** Cumulative distribution diagram of the ratio of iCLIP hits in the 3’UTR to the total number of hits. Only transcripts with at least one iCLIP hit in the 3′UTR were included. Transcripts selectively altered in axons of Tdp-43 knockdown motoneurons are labeled “axon only”

Next, we investigated the relationship between Tdp-43 binding to transcripts and their alterations in Tdp-43 knockdown motoneurons. For this purpose, we overlaid our RNA-seq datasets with the TDP-43 iCLIP data generated from human brain [18]. We found that those transcripts that were upregulated in the axonal compartment of Tdp-43 knockdown motoneurons contain a higher number of iCLIP hits than downregulated or unchanged transcripts (Fig. 4a). Since axonal transport of mRNAs is usually mediated by interactions of RNA-binding proteins with motifs in the 3′UTRs of these mRNAs [31] we next investigated the ratio of iCLIP hits in the 3′UTR compared to the total number of hits. The cumulative frequency distribution of this ratio showed that transcripts downregulated in the axonal or somatodendritic compartment of Tdp-43 knockdown motoneurons contained a higher proportion of iCLIP hits in their 3′UTRs compared to unchanged or upregulated transcripts (Fig. 4b). This effect is even more pronounced for those axonal transcripts that are selectively altered in the axonal but not somatodendritic compartment of Tdp-43 knockdown motoneurons (Fig. 4b). In contrast, transcripts upregulated in the axonal compartment upon loss of Tdp-43 showed fewer iCLIP hits in their 3′UTRs compared to unchanged or downregulated transcripts. This result suggests that Tdp-43 mediates the transport and/or stability of mRNAs in axons of motoneurons by binding to the 3′UTR of these transcripts, and it inhibits the axonal translocation when interacting with other sites.

### Loss of Tdp-43 dysregulates transcripts with mitochondrial and synapse functions in the somatodendritic compartment

**Fig. 5:**
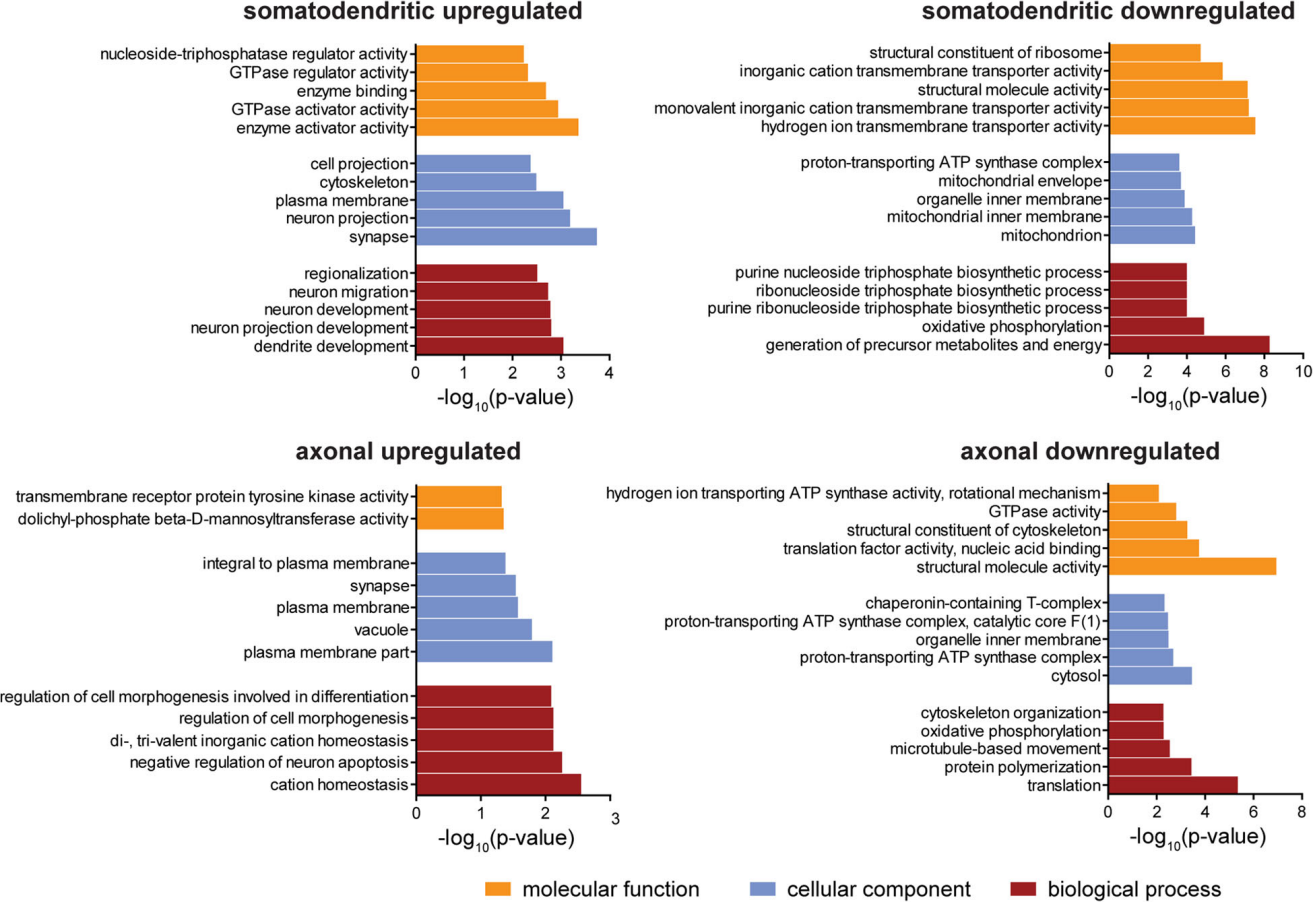
GO term enrichment analysis of transcripts dysregulated in Tdp-43 knockdown motoneurons. The top 5 GO categories for “biological process”, “cellular component” and “molecular function” for transcripts upregulated or downregulated in each compartment are shown

Gene ontology (GO) term analysis of transcripts upregulated in the somatodendritic compartment of Tdp-43 knockdown motoneurons revealed an enrichment of transcripts with functions in neuron projection development, synapse and cytoskeleton (Fig. 5). Among these is the transcript encoding Amyloid precursor protein (App) which has been found to be elevated in the SOD1G93A mouse model of ALS [32, 33]. App ablation improved the motor functions of SOD1G93A mice, indicating the functional relevance of increased APP in ALS [33]. The most strongly upregulated transcript encodes for thioredoxin interacting protein (Txnip) (Supplementary Table 3), which is an inhibitor of thioredoxin function. Thioredoxin acts as an antioxidant such that upregulation of Txnip is expected to disturb mitochondrial function and to inhibit pathways that protect neurons against reactive oxygen species (ROS) [34]. We also detected an upregulation of the *Igf1r* transcript encoding the IGF-1 receptor in agreement with previous studies reporting increased levels of IGF-1 receptor in the spinal cord of ALS patients [35, 36]. Furthermore, we found a significant upregulation of cAMP-specific 3′,5′-cyclic phosphodiesterase 4D (Pde4d), which hydrolyzes the second messenger cAMP. Reduced cAMP levels are expected to affect many functions related to neuronal survival and activity. Efforts to inhibit this enzyme have found their way into clinical trials for treatment of ALS (ClinicalTrials.gov, Identifier: NCT02238626), indicating that this upregulation could contribute to neuronal dysfunction and cell death in Tdp-43-depleted motoneurons.

In contrast, the group of transcripts downregulated in the somatodendritic compartment of Tdp-43-depleted motoneurons showed a high association with GO terms related to mitochondrial function and energy production (Fig. 5). Among the most strongly downregulated genes, we found the transcripts encoding Acetyl-CoA acetyltransferase 1 (Acat1) and Cytochrome b-c1 complex subunit 8 (Uqcrq) (Supplementary Table 4). Notably, a number of transcripts encoding proteins previously linked to ALS also appeared downregulated in Tdp-43 knockdown motoneurons including the transcripts for Sod1 [37], Hnrnpa2b1 [38] and Matr3 [39].

### Reduced capacity for protein production in axons of Tdp-43 knockdown motoneurons

**Fig. 6:**
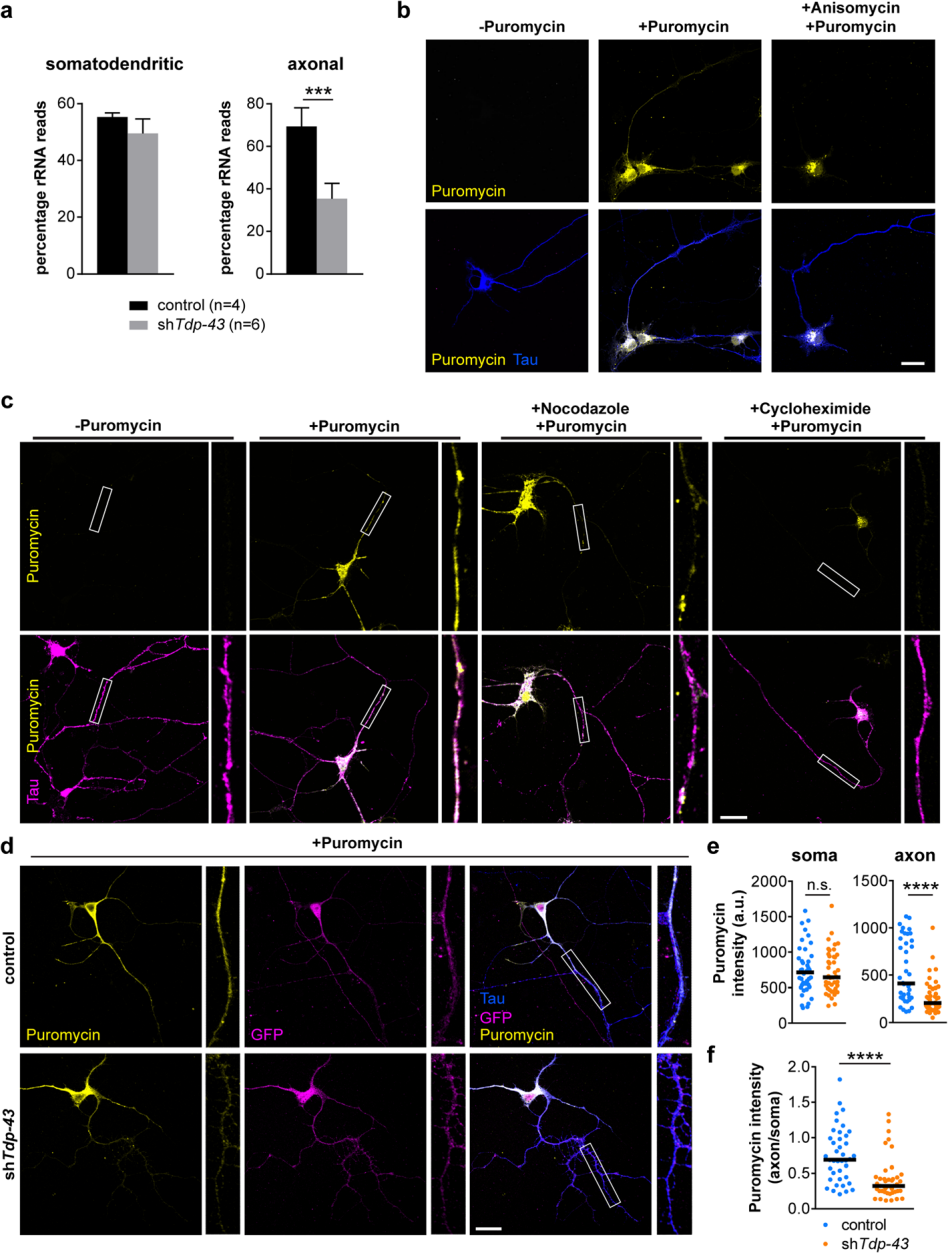
Reduced protein synthesis in axons of Tdp-43 knockdown motoneurons. **a** Percentage of reads mapping to rRNAs from RNA-seq data of Tdp-43 knockdown (sh*Tdp-43*) and control motoneurons. Data represent mean ± SD. ***p ≤ 0.001; unpaired t-test. **b** Immunostaining of cultured motoneurons with antibodies against puromycin and Tau. Motoneurons were exposed to puromycin as indicated. Treatment with anisomycin was done prior to puromycin exposure. Scale bar: 20 μm. **c** Motoneurons were exposed to nocodazole or cycloheximide as indicated followed by puromycin labeling. Immunostaining was performed with antibodies against puromycin and Tau. Boxes indicate magnified axon segments. Scale bar: 20 μm. **d** Immunostaining of control and Tdp-43 knockdown motoneurons with antibodies against puromycin and Tau. Motoneurons were transduced with control or Tdp-43 knockdown lentivirus and cultured for 6 d followed by puromycin labeling. Boxes indicate magnified axon segments. Scale bar: 20 μm. **e** Scatter plots showing fluorescence intensity of the puromycin immunosignal in somata and axons of Tdp-43 knockdown and control motoneurons. Horizontal lines represent median. n.s. not significant, ****p ≤ 0.0001; Mann-Whitney test (*N* = 40 motoneurons for control and 43 motoneurons for Tdp-43 knockdown motoneurons, *n* = 3 independent experiments). **f** Scatter plot showing the axon-to-soma ratios of puromycin intensity in control and Tdp-43 knockdown motoneurons. Horizontal lines represent median. ****p ≤ 0.0001; Mann-Whitney test (*N* = 40 motoneurons for control and 43 motoneurons for Tdp-43 knockdown motoneurons, *n* = 3 independent experiments)

For transcripts downregulated in axons of Tdp-43 knockdown motoneurons the GO term “translation” is the most significantly affected biological process (Fig. 5). The transcripts falling within this category include translation initiation factors and ribosomal proteins. For example, transcripts encoding the 40S ribosomal proteins S8 (Rps8) and S3 (Rps3) and the transcript for eukaryotic initiation factor 4A-II (Eif4a2) were among the top 20 downregulated transcripts in the axonal compartment upon Tdp-43 depletion (Supplementary Table 6). The whole transcriptome profiling method we used for RNA-seq also monitors non-coding RNAs including rRNAs [22]. We found a significant reduction in the number of reads mapping to rRNA in axons of Tdp-43 knockdown motoneurons compared to controls while rRNA levels in the somatodendritic compartment were unchanged (Fig. 6a). Based on these results we investigated the possibility that Tdp-43 deficiency impairs protein translation in axons. We set up a puromycin labeling assay in which motoneurons were cultured for 6 d and then exposed to puromycin for 15 min. Puromycin is incorporated into nascent polypeptide chains and can be detected by immunostaining with a puromycin-specific antibody. Following puromycin exposure, wildtype motoneurons showed prominent immunolabeling in the cytosol of the soma as well as in axons (Fig. 6b). Omission of puromycin or pre-treatment with the protein synthesis inhibitor anisomycin strongly reduced the immunosignal, indicating specificity of the puromycin labeling assay. The axonally localized puromycin immunoreactivity most likely originated from proteins that were locally synthesized in axons, because the 15 min pulse is too short to allow anterograde transport of these proteins from the cell body. To test this possibility in more detail, we exposed motoneurons to nocodazole, an inhibitor of microtubule assembly that disrupts axonal transport [40], prior to puromycin labeling. Following nocodazole treatment, puromycin immunoreactivity was still detectable in axons of motoneurons (Fig. 6c). Treatment with the translation inhibitor cycloheximide prior to puromycin exposure abolished puromycin immunolabeling indicating signal specificity. Thus, puromycin immunoreactivity in axons of motoneurons reflects local protein synthesis that can occur in the absence of anterograde axonal transport. Next, we investigated puromycin incorporation in Tdp-43 knockdown motoneurons (Fig. 6d). This analysis showed that puromycin immunolabeling was exclusively reduced in axons but not in somata of Tdp-43 knockdown motoneurons relative to controls (Fig. 6e) resulting in a significantly reduced ratio of axonal to somatic puromycin labeling intensity upon Tdp-43 depletion (Fig. 6f). Thus, Tdp-43-depleted motoneurons have a reduced capacity for protein synthesis in their axons.

### Disturbed mitochondrial functionality in Tdp-43-deficient axons

**Fig. 7:**
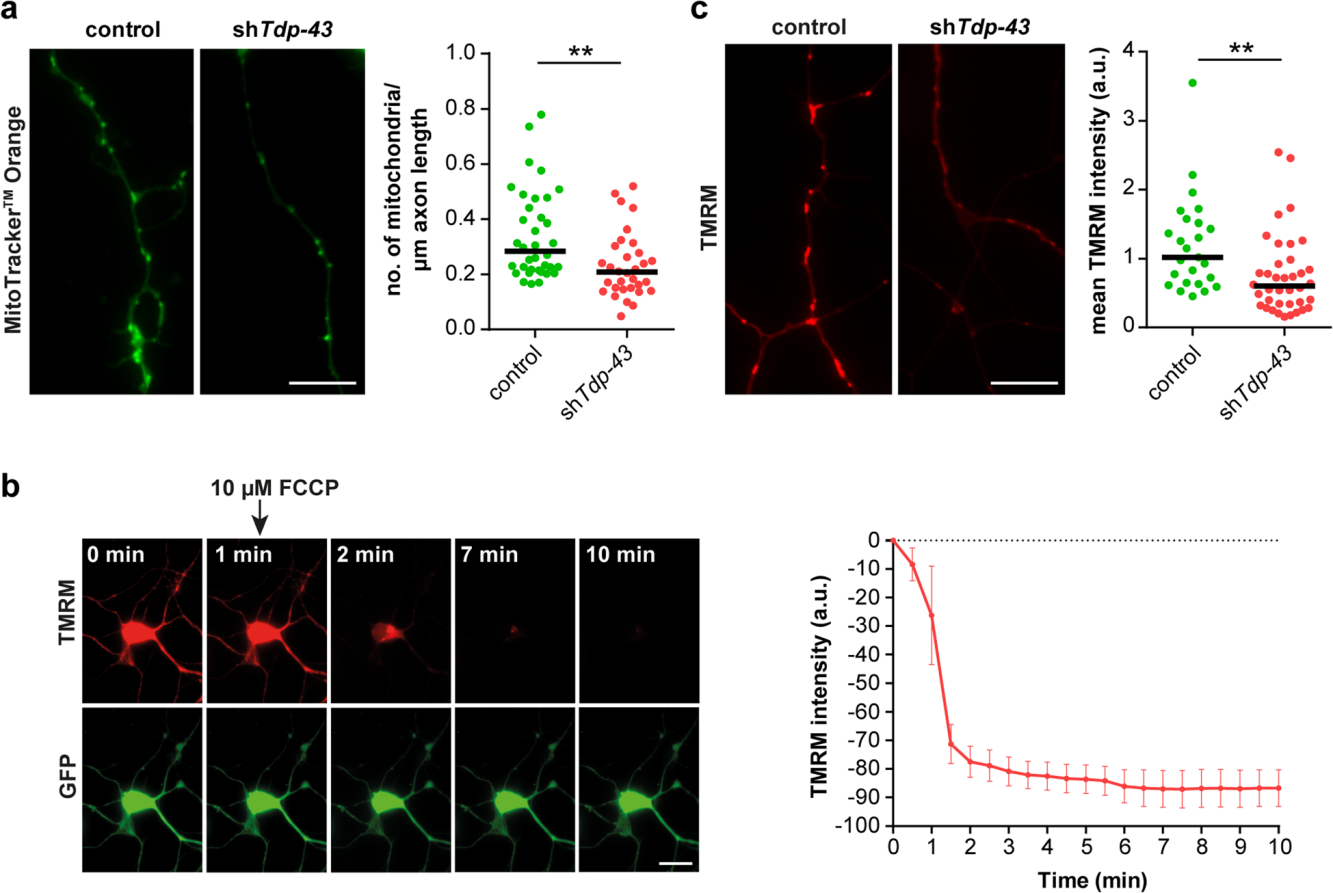
Mitochondrial dysfunction in axons of Tdp-43 knockdown motoneurons. **a** Left panel: Axons of control and Tdp-43 knockdown (sh*Tdp-43*) motoneurons stained with MitoTracker™ Orange CM-H2TMRos. Scale bar: 5 μm. Right panel: Scatter plot depicting the number of mitochondria stained with MitoTracker™ Orange CM-H2TMRos normalized to axon length. Horizontal lines represent median. **p ≤ 0.01; Mann-Whitney test (*N* = 36 axons for control and 32 axons for Tdp-43 knockdown motoneurons, *n* = 2 independent experiments). **b** Left panel: Motoneurons transduced with control lentivirus (expressing GFP) were labeled with TMRM for live-cell imaging. Cells were treated with FCCP at the indicated timepoint to disrupt the mitochondrial membrane potential. Scale bar: 20 μm. Right panel: Quantification of TMRM intensity in mitochondria with time. Data represent mean ± SD (*n* = 5 mitochondria). **c** Left panel: Axons of control and Tdp-43 knockdown motoneurons labeled with TMRM. Scale bar: 5 μm. Right panel: Quantification of TMRM mean intensity of mitochondria in axons of control and Tdp-43 knockdown motoneurons. Horizontal lines represent median. **p ≤ 0.01; Mann-Whitney test (*N* = 25 axons for control and 38 axons for Tdp-43 knockdown motoneurons, *n* = 3 independent experiments)

Beyond transcripts related to translation, we found an enrichment of GO terms related to mitochondrial functions for transcripts downregulated in the axonal compartment of Tdp-43 knockdown motoneurons (Fig. 5). These GO terms contain the transcript encoding the mitochondrial ATP synthase beta-subunit (Atp5b) which has previously been associated with ALS [41]. In order to assess whether axonal mitochondria are affected by Tdp-43 depletion, we labeled mitochondria in control and Tdp-43 knockdown motoneurons with MitoTracker™ Orange. We found that the number of intact mitochondria was significantly reduced in axons of Tdp-43 knockdown motoneurons relative to controls (Fig. 7a). Next, we assessed the mitochondrial membrane potential using the fluorescent dye tetramethylrhodamine (TMRM). As a negative control we used the uncoupling agent FCCP, which depolarizes the mitochondrial membrane potential. Treatment with FCCP led to rapid loss of the TMRM signal indicating the specificity of the labeling procedure (Fig. 7b). We then labeled control and Tdp-43 knockdown motoneurons with TMRM (Fig. 7c). This analysis revealed that the mean labeling intensity of mitochondria was significantly reduced in axons of Tdp-43 knockdown motoneurons relative to controls. Thus, Tdp-43 depletion affects the number and functionality of axonal mitochondria.

### Nicotinamide treatment rescues axonal defects of Tdp-43-deficient motoneurons

**Fig. 8:**
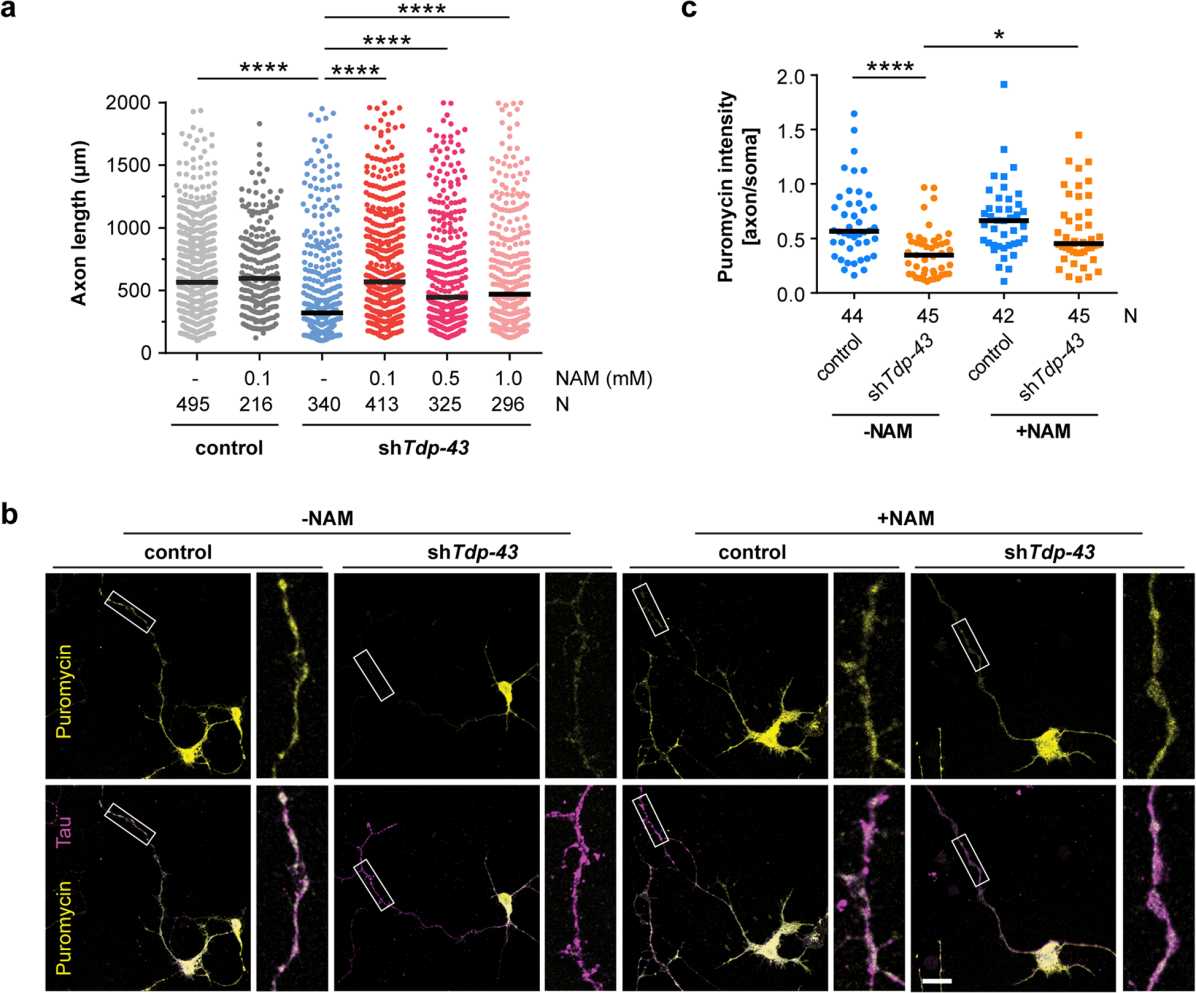
Nicotinamide (NAM) rescues axon defects of Tdp-43 knockdown motoneurons. **a** Scatter plot of axon lengths of Tdp-43 knockdown (sh*Tdp-43*) and control motoneurons cultured in the absence (−) or presence of NAM at the indicated concentrations. Horizontal lines represent median. ****p ≤ 0.0001; Kruskal-Wallis test with Dunn’s multiple comparisons test (N numbers of axons are shown in the graph, *n* = 3 independent experiments). **b** Immunostaining of control and Tdp-43 knockdown motoneurons with antibodies against puromycin and Tau. Motoneurons were transduced with control or Tdp-43 knockdown lentivirus and cultured in the presence (+) or absence (−) of 0.1 mM NAM. Puromycin labeling was performed at DIV6. Scale bar: 20 μm. **c** Scatter plot showing the axon-to-soma ratios of puromycin intensity in control and Tdp-43 knockdown motoneurons. Horizontal lines represent median. *p ≤ 0.05, ****p ≤ 0.0001; Kruskal Wallis test with Dunn’s multiple comparisons test (N numbers of motoneurons are shown in the graph, *n* = 3 independent experiments)

Our data point towards the possibility that a lack of energy supply due to disturbed mitochondrial function contributes to the axon growth defect associated with Tdp-43 depletion in motoneurons. To investigate this possibility we treated Tdp-43 knockdown and control motoneurons with nicotinamide (NAM), a precursor for nicotinamide adenine dinucleotide (NAD+) that acts as a key cofactor in mitochondrial pathways for the production of ATP. NAM is converted to NAD+ through the salvage pathway [42]. We found that treatment with 0.1 mM NAM restored axon growth of Tdp-43 knockdown motoneurons to normal levels, while axons of control motoneurons were not altered by exposure to NAM (Fig. 8a). The same rescue effect was observed with NAM concentrations up to 1 mM.

We then tested whether NAM treatment had an impact on the defective axonal protein synthesis which we observed upon Tdp-43 depletion. Control and Tdp-43 knockdown motoneurons were cultured in the presence or absence of 0.1 mM NAM for 6 d followed by puromycin exposure to label nascent proteins (Fig. 8b). In the absence of NAM, puromycin immunolabeling was reduced in axons of Tdp-43 knockdown motoneurons relative to control motoneurons (Fig. 8b, c). Treatment with NAM significantly increased puromycin immunoreactivity in axons of Tdp-43 knockdown motoneurons relative to axons of untreated Tdp-43 knockdown motoneurons (Fig. 8b, c). Thus, NAM exposure rescued the axon growth defect of motoneurons depleted of Tdp-43 and increased their capacity for axonal protein synthesis.

## Discussion

In this study, we have investigated transcriptome alterations in the somatodendritic and axonal compartments of motoneurons after Tdp-43 depletion. Subcellular transcriptome alterations in ALS cell models have been investigated before using different approaches. In the study by Rotem et al. mouse motoneurons were transduced with lentiviruses for expression of GFP-tagged SOD1(G93A) or TDP-43(A315T) and cultured in modified Boyden chambers to obtain neuritic RNA [43]. Nijssen et al. developed an optimized procedure for conducting axonal RNA-seq on motoneurons grown in microfluidic chambers and used this technique to profile axons of mouse embryonic stem cell (mESC)-derived motoneurons overexpressing SOD1(G93A) [44]. While these studies revealed candidate transcripts whose dysregulation might contribute to motoneuron dysfunction in ALS, it needs to be considered that overexpression of SOD1 or TDP-43 might induce deleterious side effects in a dose-dependent manner [45].

It has been suggested that, in addition to the toxicity exerted by TDP-43 insoluble inclusions, loss of TDP-43 functions contributes to the etiology of ALS [46]. Given that TDP-43 ALS mutants show reduced motility in axons [4] and that axonal degeneration precedes motoneuron loss in ALS mouse models [47, 48], loss of axonal TDP-43 functions might be an early driving factor contributing to the motoneuron pathology underlying ALS. Therefore, to investigate the effects of Tdp-43 depletion on the composition of subcellular transcriptomes we cultured Tdp-43 knockdown motoneurons in microfluidic chambers and used a whole transcriptome profiling method for identification of RNA alterations. Major changes were detectable in the somatodendritic as well as the axonal compartment and similar numbers of transcripts were up- or downregulated. These deregulations might disturb multiple pathways and mechanisms that are relevant for neuronal survival and axonal maintenance as well as for synaptic function and integrity. In agreement with this notion, we observed several defects in axons of Tdp-43-deficient motoneurons, including loss of transcripts encoding cytoskeletal proteins, reduced protein synthesis and disturbed mitochondrial function. In addition to transcripts encoding proteins important for motoneuron biology, we observed that the axonal localization also of non-polyadenylated RNAs such as ribosomal RNAs is affected by Tdp-43 depletion. Therefore, our results suggest that the pathology induced by Tdp-43 deficiency cannot simply be explained by one individual key mechanism, but rather by a combination of defects that play together and contribute to a complex disease phenotype.

After Tdp-43 depletion, several transcripts are upregulated in the soma that appear highly relevant for neuronal integrity. For example, upregulation of Txnip is expected to result in an inhibition of thioredoxin leading to enhanced oxidative stress. The Pi3K/AKT pathway, which is important for neuronal survival [49] and proper mitochondrial function [50], modulates Txnip expression and thereby protects cells against ROS [51]. AKT-mediated inhibition of the transcription factor FOXO1 can reduce Txnip levels [51]. This then leads to enhanced thioredoxin activity, which is necessary for ROS detoxification. The observations that FOXO1 represses *TXNIP* transcription directly [52, 53], and that TDP-43 controls FOXO activity [54] indicate that this pathway might contribute to ALS pathology. The enhanced expression of *Txnip* we observed after Tdp-43 depletion could cause inhibition of thioredoxin activity, leading to enhanced ROS levels. This, in turn, could induce the observed mitochondrial dysfunction and thus contribute to the pathological alterations in mitochondria seen in Tdp-43-associated motoneuron disease models. The rescue effect by treatment with NAM, which has been shown to reduce ROS levels [55], supports this hypothesis and indicates that this pathway could represent a central part of the pathology in ALS and other diseases in which TDP-43 loss of function is apparent.

GO term analysis of transcripts downregulated in the axonal compartment of Tdp-43-deficient motoneurons revealed that pathways related to mitochondrial functions might be particularly affected. Mitochondria are a known target of Tdp-43 function [26, 27, 56–60] and are also defective in iPS-cell derived motoneurons harboring ALS mutations [61] and ALS mouse models [62], indicating that defects in energy production upon loss of Tdp-43 might contribute to the axonal growth defect in cultured motoneurons. In agreement with this notion, we found a decreased number and activity of mitochondria in axons of Tdp-43 knockdown motoneurons. Moreover, treatment with the NAD+ precursor NAM restored axon growth of Tdp-43-depleted motoneurons to near-normal levels. This suggests that reduced energy levels might play an important role in the axonal pathology upon Tdp-43 loss. However, besides energy support, NAM can also exert regulatory functions outside mitochondria. For example, Sirtuins (SIRTs) are protein deacetylases that use NAD+ as a cofactor and convert it to NAM during the deacetylation reaction [63]. NAM itself acts as an inhibitor of Sirtuin activities. Inhibition of SIRT2 has been found to be neuroprotective in models of Parkinson’s disease and other neurodegenerative disease contexts [64, 65]. Therefore, dysregulation of Sirtuins might contribute to the axonal defects we observed in Tdp-43 knockdown motoneurons. Recently, the administration of NAM has been shown to improve motor symptoms of SOD1G93A mice [66]. Furthermore, reduced NAM levels have been observed in ALS patients [66]. Thus, NAM and associated downstream pathways might represent potential avenues for new ALS treatment options.

We found that reduced axon growth is a key morphological phenotype of Tdp-43-deficient motoneurons. We observed this phenotype with three different shRNAs targeting the *Tdp-43* transcript. The reduced axon lengths we observed for Tdp-43-depleted motoneurons stand in contrast to previous observations [25], but our data are in agreement with other studies investigating neuronal defects upon TDP-43 reduction [67, 68]. Beyond that, expression of TDP-43 mutants harboring ALS mutations also leads to neurite growth defects [69]. Reduced axon growth is seen in many pathologies relevant to motoneuron diseases. In the case of SMN protein deficiency which causes SMA, reduced axon growth accompanied by deficits in presynaptic actin synthesis and excitability were observed in mouse models [70–73]. In motoneurons depleted of C9orf72 as a cell culture model of ALS, dysregulation of actin dynamics in axons appears prominent [74]. This raises the question whether similar or distinct mechanisms are responsible for defective axon growth in Tdp-43-depleted motoneurons. Our data show that multiple pathways are affected by loss of Tdp-43. On the one side, mitochondrial dysfunction and enhanced ROS levels are expected to reduce cellular metabolism and thus also axon growth. On the other side, we found dysregulation of transcripts related to protein translation in the axonal compartment of Tdp-43 knockdown motoneurons. Also, rRNA levels were significantly reduced in the axonal but not somatodendritic compartment of Tdp-43 knockdown motoneurons. In agreement, we found that Tdp-43-depleted motoneurons have a reduced capacity for axonal protein synthesis, which might contribute to defective axon growth. Treatment with NAM partially rescued this defect, indicating that pathways related to energy metabolism can alleviate or delay pathological processes in axons of motoneurons with loss of Tdp-43 function.

In summary, our data have revealed that multiple pathways are dysregulated in Tdp-43-depleted motoneurons, many of them having been characterized before in the context of other neurodegenerative diseases such as Alzheimer’s, Huntington’s and Parkinson’s disease. Thus, it might be the combination of defects in these pathways that underlies the vulnerability and axonal degeneration of Tdp-43-deficient motoneurons. A multitude of targets could be conferred from these pathways that might need to be addressed in combination for therapy development in ALS.

## Supplementary information

**Additional file 1: Supplementary Table 1.** List of primers for cloning of shRNAs into pSIH. siRNA sequences are underlined. **Supplementary Table 2.** List of qPCR primers. **Supplementary Table 3.** List of transcripts significantly (*p*<0.05) upregulated in the somatodendritic compartment of Tdp-43 knockdown (T_sd) motoneurons relative to control (G_sd) motoneurons. **Supplementary Table 4.** List of transcripts significantly (*p*<0.05) downregulated in the somatodendritic compartment of Tdp-43 knockdown (T_sd) motoneurons relative to control (G_sd) motoneurons. **Supplementary Table 5.** List of transcripts significantly (*p*<0.05) upregulated in the axonal compartment of Tdp-43 knockdown (T_ax) motoneurons relative to control (G_ax) motoneurons. **Supplementary Table 6.** List of transcripts significantly (*p*<0.05) downregulated in the axonal compartment of Tdp-43 knockdown (T_ax) motoneurons relative to control (G_ax) motoneurons. **Supplementary Table 7.** List of transcripts significantly (*p*<0.05) deregulated in the somatodendritic (sd) and axonal (ax) compartment of Tdp-43 knockdown motoneurons relative to control motoneurons.

## Data Availability

The sequencing data described in this publication are accessible in NCBI’s Gene Expression Omnibus through GEO Series accession number GSE147607.
